# Regulation of transient receptor potential channels by traditional Chinese medicines and their active ingredients

**DOI:** 10.3389/fphar.2022.1039412

**Published:** 2022-10-13

**Authors:** Shidu Yan, Yuchan Huang, Qian Xiao, Zixia Su, Lei Xia, Jinling Xie, Fan Zhang, Zhengcai Du, Xiaotao Hou, Jiagang Deng, Erwei Hao

**Affiliations:** ^1^ Guangxi Key Laboratory of Efficacy Study on Chinese Materia Medica, Guangxi University of Chinese Medicine, Nanning, Guangxi, China; ^2^ Guangxi Collaborative Innovation Center of Research on Functional Ingredients of Agricultural Residues, Guangxi University of Chinese Medicine, Nanning, Guangxi, China; ^3^ Guangxi Key Laboratory of TCM Formulas Theory and Transformation for Damp Diseases, Guangxi University of Chinese Medicine, Nanning, Guangxi, China

**Keywords:** traditional Chinese medicine, Chinese herbal medicinal properties, transient receptor potential channels, natural compounds, pain relief

## Abstract

In recent years, activation of thermal transient receptor potential (TRP) ion channels at a range of temperatures has received widespread attention as a target for traditional Chinese medicine (TCM) to regulate body temperature and relieve pain. Discovery of transient receptor potential vanilloid 1 (TRPV1) was awarded a Nobel Prize, reflecting the importance of these channels. Here, the regulatory effects of TCMs and their active ingredients on TRP ion channels are reviewed, and future directions for research on the cold, hot, warm, cool, and neutral natures of TCMs are considered. In herbs with cold, hot, warm, cool, and neutral natures, we found 29 TCMs with regulatory effects on TRP ion channels, including *Cinnamomi Cortex*, *Capsici Fructus*, *Rhei Radix et Rhizoma*, *Macleayae cordatae Herba*, *Menthae Haplocalycis Herba*, and *Rhodiolae Crenulatae Radix et Rhizoma*. Although some progress has been made in understanding the regulation of TRP ion channels by TCMs and their ingredients, the molecular mechanism by which TCMs have this effect remains to be further studied. We hope this review will provide a reference for further research on the cold, hot, warm, cool, and neutral natures of TCMs.

## 1 Introduction

Traditional Chinese Medicine (TCM) is a common clinical treatment in traditional Chinese Medicine, and it has demonstrated efficacy in the treatment of complex diseases and major epidemics. Each TCM is a complex medicine with synergy between multiple ingredients, targets, and pathways, and each ingredient has the potential to develop into a new drug. The property theory of TCM is an accumulation of long-term clinical application of TCM, and is an important basis for TCM use. In addition, the four qi theory has important guiding significance for rational use of TCM in clinic (Ma et al., 2020; Zhu et al., 2021) The transient receptor potential (TRP) family mediates the sensations of heat, cold and pain and an understanding of its function can in turn enhance understanding of the pathophysiological regulation of body temperature or pain relief. It is also one of the molecular bases for an in-depth understanding of the cold-hot theory of TCM, and it provides a basis for studying the cold-hot natures of TCM (Gao et al., 2014; Hung and Tan, 2018; Viana, 2018; Isaacson and Hoon, 2021).

Currently, there is a lack of reviews on the regulation of TRP channels by various types of TCMs, and an updated review may help to demonstrate gaps in research, which may also provide new ideas for more in-depth studies in the future. This review focuses on TCMs with regulatory effects on TRP ion channels to systematically explore the molecular mechanism of TCM exerting cold, hot, warm, cool and neutral nature. It will provide a reference for the more scientific use of various types of traditional Chinese medicine in the future.

## 2 Transient receptor potential channels

Transient receptor potential channels, a class of ion channel that can be activated within a specific temperature range, were discovered in *Drosophila melanogaster*. TRP ion channels are found in many tissues and have a wide range of physiological functions related to vision, smell, taste, hearing, touch, and body temperature (Uchida et al., 2017). Seven families of TRPs have been identified in mammals, including the vanilloid receptor family (TRPV), the TRP melastatin (TRPM), the TRP ankyrin (TRPA), the TRP canonical (TRPC), the TRP polycystin (TRPP), the TRP mucolipin (TRPML), the TRP NomPC-like (TRPN) (Li, 2017).

**FIGURE 1:**
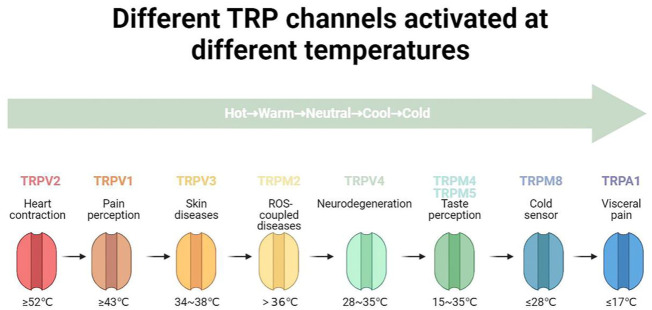
Different TRP channels activated at different temperatures.

TRPC1-7 are activated in a G-protein-coupled receptor-phospholipase C-dependent manner, which is similar to the TRPs of *Drosophila* (Chen et al., 2020). Mutations in TRPPs cause autosomal dominant polycystic kidney disease and are also involved in taste perception and fertilization processes (Su et al., 2018). TRPML1-3 mediate endo-lysosomal functions such as autophagy, pH regulation and lysosomal exocytosis that are closely related to cancer development (Jaślan et al., 2020; Xu and Dong, 2021). TRPN contributes to the adaption of mechanosensory transduction currents such as tactile sense, auditory, and proprioception, independent of their ion-conduction function (Jin et al., 2017; Li et al., 2021). Three of these families, the TRP vanilloid (TRPV), the TRP melastatin (TRPM), and TRP ankyrin (TRPA), have received a great deal of attention due to their association with cold and heat sensation. The different TRP channel families share common structural features, such as six putative transmembrane (TM) spanning domains with intracellular C and N termini, and a pore lining between the fifth (S5) and sixth (S6) TM. TRP channels function as cellular sensors, regulation of intracellular calcium signalling/free calcium concentration in many cases, and play an important role in muscle contraction, cell differentiation and proliferation, gene transcription, and many other cellular functions as well as cell death (Clapham et al., 2005; Bradshaw et al., 2013; Li, 2017). Jaffal et al. proposed that TRP channels may be involved in the pain, inflammation, fever, and other complications associated with COVID-19, and that blocking or inhibiting TRP channels may be a means of preventing and treating this disease (Jaffal and Abbas, 2021). The relevant functional information of TRP channels that can be activated by different temperatures are comprehensively summarized in Figure 1 and Table 1.

**TABLE 1:** Introduction to TRP channels

Channel	Temperature sensitivity	Main tissue distribution	Functions	References
TRPV1	≥43°C	Sensory neurons, brain, skin.	Noxious thermoreceptors; also involved in inflammatory pain, thermal nociceptive hypersensitivity, hippocampal chronic depression, obesity, diabetes, bladder function, hypertension, hypothermia, gastroenteritis, renal excretory function	(Vay et al., 2012; Uchida et al., 2017; Wu, 2019)
TRPV2	≥52°C	Sensory neurons, brain, spinal cord, lung, liver, spleen, colon, heart, immunocyte.	Extreme temperature sensor; innate immune system	(Kochukov et al., 2006; Vay et al., 2012; Naticchioni et al., 2015; Uchida et al., 2017)
TRPV3	34-38°C	Sensory neurons, skin, brain, spinal cord, stomach, colon.	Warmth receptors; may be associated with detection of harmful heat levels	(Kochukov et al., 2006; Vay et al., 2012; Uchida et al., 2017; Neuberger et al., 2021a)
TRPV4	28-35°C	Sensory neurons, skin, brain, kidneys, lungs, inner ear, bladder.	Thermoreceptors; may be associated with noxious mechanical pain and thermal nociceptive sensitization	(Vay et al., 2012; Uchida et al., 2017; Huang et al., 2019)
TRPM2	>36°C	Brain, immunocyte, pancreas.	Thermoreceptors; associated with pain due to inflammation, diabetes, tumors, cardiovascular system	(Kahya et al., 2017; Uchida et al., 2017)
TRPM4	15-35°C	Heart, liver, immunocyte, pancreas.	Thermoreceptors; associated with inflammation-induced pain, tumors, cardiovascular system	(Talavera et al., 2005; Uchida et al., 2017; Dutta Banik et al., 2018)
TRPM5	15-35°C	Taste cells, pancreas.	Warmth receptors; may be related to insulin secretion, obesity, diabetes	(Talavera et al., 2005; Uchida et al., 2017; Dutta Banik et al., 2018)
TRPM8	≤28°C	Sensory neurons, bladder.	Nontoxic cold sensory receptors, behavioral thermoregulation, cold-mediated analgesia; cold injurious sensation in some neurons	(Vay et al., 2012; Uchida et al., 2017; Yin et al., 2018)
TRPA1	≤17°C	Sensory neurons, heart, lungs, brain, pancreas, gastrointestinal tract, bladder.	Cold, mechanically- and chemically-induced injuries, cold nociceptive sensitization	(Vay et al., 2012; Uchida et al., 2017

### 2.1 Classification of transient receptor potential channels

#### 2.1.1 Transient receptor potential vanilloid 1

TRPV1 is a non-selective ligand-gated cation channel, which in humans functions as a pain sensor under thermal stimulation (Caterina et al., 1997). The normal activation threshold of TRPV1 was found to be ≥43°C, while conditions such as acidic environment (pH<5.0), voltage stimulation, capsaicin, osmotic concentration, and endogenous cannabinoids can also activate the channel. In response, the body may produce a combination of sensations such as acidity, heat, burning, pain, and itching (Liu et al., 2017b; Wu, 2019). Julius et al. found a loss of sensitivity to capsaicin and resiniferatoxin in TRPV1^−/−^ mice *via* various cellular and mouse behavioral analyses (Caterina et al., 2000). Lubova et al. found that K688G as a part of the gating apparatus of the TRPV1 channel may uncouple the TRP structural domain from its pore, thus leading to spontaneous activation and desensitization of the channel, which provides a basis for further study of this channel (Lubova et al., 2020).

#### 2.1.2 Transient receptor potential vanilloid 2

TRPV2 is a calcium-permeable, non-selective cation channel that is essential for normal cardiac contractility and is widely distributed in central and peripheral nerves, including the spinal cord, brain, dorsal root ganglia, and trigeminal ganglion (Naticchioni et al., 2015; Xue et al., 2021). Using immunohistochemistry, Nedungadi et al. found that TRPV2 channels are abundantly expressed in the giant cell division of the anterior brain, supraoptic nucleus, and paraventricular nucleus of the hypothalamus (Nedungadi et al., 2012). The thermal activation threshold of TRPV2 is as high as 52°C (Kochukov et al., 2006).

#### 2.1.3 Transient receptor potential vanilloid 3

TRPV3 is activated in the temperature range from 34°C to 38°C and is involved in a variety of physiological processes, such as temperature perception, nociception, pruritus, and wound healing (Kochukov et al., 2006; Neuberger et al., 2021a). Deng et al. found that S100A4 (S100 calcium-binding protein family) interacts with the TRPV3 channel protein, thereby significantly enhancing the latter’s ability to regulate calcium ions, with implications for the treatment of neurological diseases (Deng et al., 2021).

#### 2.1.4 Transient receptor potential vanilloid 4

TRPV4 is activated in the temperature range from 28°C to 35°C (Huang et al., 2019) and can directly cause hereditary neurodegenerative syndromes. Woolums et al. found that TRPV4-mediated neurotoxicity is regulated by the Ca^2+^-binding mitochondrial GTPase Miro, which demonstrates that TRPV4 antagonists are effective in the treatment of TRPV4-mediated neurodegenerative diseases (Woolums et al., 2020).

#### 2.1.5 Transient receptor potential melastatin 2

TRPM2 plays an important role in the sensation of warmth and helps to control body temperature. TRPM2^−/−^ results in a loss of thermal detection capability (Paricio-Montesinos et al., 2020). Tan and McNaughton. (2016) found that at temperatures above 23°C TRPM2 expressed in somatosensory neurons triggers signals which generate a sensation of warmth in mice, causing them to look for cooler environments. TRPM2^−/−^ mice were unable to distinguish between cool and warmth. Kashio et al. (2012) used the [Ca^2+^]_i_ test to analyze the temperature threshold for activation of TRPM2 and found that temperature of about 47°C can also activate TRPM2. Gattkowski et al. discovered a novel CaM-binding site in the NudT9H domain of TRPM2, which may have a role in the temperature sensitivity of TRPM2 channels. The discovery of this site laid the foundation for the study of temperature-activated TRPM2 channels (Gattkowski et al., 2019).

#### 2.1.6 Transient receptor potential melastatin 4 and transient receptor potential melastatin 5

TRMP4 and TRPM5 are thermally activated ion channels which are highly sensitive to temperatures ranging from 15°C to 35°C (Talavera et al., 2005). Using live-cell imaging and behavioral analysis of knockout mice, Dutta Banik et al. found that TRPM4 and TRPM5 are required for transduction of gustatory stimulation and loss of either channel severely affects taste perception (Dutta Banik et al., 2018).

#### 2.1.7 Transient receptor potential melastatin 8

TRPM8 is the body’s primary sensor of cold and menthol perception, can be activated by temperatures below 28°C, and is a relevant target for the treatment of pain, such as chronic pain and migraine (Yin et al., 2018). Huang et al. demonstrated that the K423 in the N-terminal of TRPM8 was the major ubiquitination residue for the regulation of TRPM8 by tripartite motif-containing 4 (TRIM4), laying the foundation for study of the interaction between TRIM4 and TRPM8 (Huang et al., 2021).

#### 2.1.8 Transient receptor potential ankyrin 1

TRPA1, a tetrameric, nonselective cation channel in dorsal root sensory neurons, is a key factor in mediating visceral pain. TRPA1 channel agonists can treat obesity and other related diseases, such as type 2 diabetes, by regulating the secretion of insulin, providing a new avenue for study on the treatment of diabetes (Liu et al., 2019; Sahin et al., 2019). TRPA1 is a low threshold sensor (≤17°C). Zhao et al. investigated a highly conserved calcium-binding pocket which can explain all aspects of calcium-dependent TRPA1 regulation, including activation, enhancement, and desensitization of metabolic receptors (Zhao et al., 2020).

## 3 Studies on regulation of transient receptor potential channels by hot traditional Chinese medicines and their components

### 3.1 Capsici Fructus

Capsici Fructus is the dried fruit of *Capsicum annuum* L, pertaining to Solanaceae, which is acrid and hot in nature (State Pharmacopoeia Commission, 2020). Julius et al. investigated the phenomenon of pain desensitization due to the long-term use of capsaicin (CAP). They isolated a functional cDNA encoding capsaicin receptor from sensory neurons using an expression cloning approach based on calcium influx. Using this approach, they found that VR1 (an early name for TRPV1) can be activated by a range of toxic temperatures, and TRPV1 became known as the capsaicin receptor (Caterina et al., 1997). CAP can induce intracellular kinases, including PLC, PKCε, PKA, and TrkA, to lower the thermal threshold of TRPV1 and thus search for novel analgesics through phosphorylated the TRPV1 channels (Szolcsányi, 2015). In addition, CAP is thermogenic in nature and activates brown adipose tissue (BAT) for fat loss by acting on TRPV1 channels (Saito, 2015). Through a series of animal model experiments, Liang et al. concluded that CAP regulates the expression levels of genes related to glucose metabolism, such as PPARα, GLUTs, IRS-1&2, PI3K, AKT, UCPs, and PDX-1, by activating TRPV1 channels. This finding demonstrated that CAP may activate the TRPV1 channel to direct insulin signaling in the brain and treat diabetes and obesity by lowering blood sugar and improving insulin sensitivity (Liang et al., 2021).

### 3.2 Cinnamomi Cortex

Cinnamomi Cortex is the bark of *Cinnamomum cassia* Presl, pertaining to Lauraceae, which is acrid, sweet, and hot in nature (Ranasinghe et al., 2013; State Pharmacopoeia Commission, 2020; Zhang and Ye, 2020). Bhave et al. demonstrated that cAMP-dependent protein kinase (PKA) directly phosphorylates VR1 (TRPV1) and increased VR1 sensitivity (Bhave et al., 2002). Li et al. found that Cinnamomi Cortex can increase the levels of cAMP and cGMP, and can significantly increase the ratio of cAMP/cGMP. Its ingredient cinnamaldehyde (CA) can significantly affect the TRP channel of dorsal root ganglion (DRG) neurons after cold load, and can thereby up-regulate the expression of TRPV1 mRNA and down-regulate the expression of TRPM8 mRNA (Sui et al., 2010; Li et al., 2016). Experiments by Hynkova et al. (2016) showed that CA can be used to study the molecular mechanisms regulating TRPA1 channels, such as the N-terminal tetrapeptide T/SPLH motifs. Kang et al. also demonstrated that the concentration of Ca^2+^ in the cytoplasm was reduced under cold stress, while the cold-sensitive channel TRPA1 was opened. CA was found to promote the excitation of cold-sensitive channel TRPA1 and return Ca^2+^ to the cytoplasm, thereby increasing the concentration of Ca^2+^ and activating cell function, thus demonstrating the warming effect of Cinnamomi Cortex (Kang et al., 2017).

### 3.3 Evodiae/Euodiae Fructus

Evodiae Fructus is the almost ripe fruit of *Evodia rutaecarpa* (Juss.) Benth, *Evodia rutaecarpa* (Juss.) Benth. var. *officinalis* (Dode) Huang or *Evodia rutaecarpa* (Juss.) Benth. var. *bodinieri* (Dode) Huang, pertaining to Rutaceae, which is acrid, bitter, hot, and mildly toxic in nature (State Pharmacopoeia Commission, 2020; Zhang and Ye, 2020). Iwaoka et al. used calcium imaging, whole-cell patch-clamp technology, and behavioral analysis in rats to find that *in vivo* treatment with evodiamine could inhibit the thermal hyperalgesia induced by plantar injection of capsaicin, and that capsazepine, a competitive antagonist of TRPV1, can completely block the effect of evodiamine (Iwaoka et al., 2016). In contrast, Liu et al. (2022) verified that evodiamine could mediate reactive oxygen species-dependent cytotoxicity associated with the TRPV1/Ca^2+^ pathway. A study by Wang et al. provides evidence that evodiamine and rutaecarpine may be partial agonists (antagonists) of TRPV1 since they may share binding sites with capsaicin (Wang et al., 2016). Through experiments in rats, Li et al. (2018) demonstrated that evodiamine could agonize the TRPA1/CGRP signaling pathway, thereby inhibiting the gastric mucosal damage caused by cold-binding stress (Li et al., (2020). Through TRPV1, Liu demonstrated that the combination of Evodiae Fructus and Zingiberis Rhizoma Recens can exceed the maximum effect that can be achieved when they are used alone (Liu, 2017).

### 3.4 Zingiberis Rhizoma

Zingiberis Rhizoma is the rhizoma of *Zingiber officinale* Rosc, pertaining to Zingiberaceae, which is acrid and hot in nature (State Pharmacopoeia Commission, 2020; Zhang and Ye, 2020). Li et al. (2018) found that Zingiberis Rhizoma may increase the expression of TRPV1 gene and protein and decrease the expression of TRPM8 gene and protein by up-regulating the cAMP/PKA pathway in primary rat dorsal root ganglion cells, thus exhibiting the hot nature of Zingiberis Rhizoma (Li et al., 2018). T551 and E571 are key sites for the binding of 6-gingerol, 6-Shogaol and zingerone to the TRPV1 channel (Yin et al., 2019a). Yang indicated that the hot nature of Zingiberis Rhizoma is achieved by activating the TRPV1 channels, and its ingredients 6- and 10-gingerol can release catecholamines to produce a systemic warm sensation, but this process can be counteracted by TRPV1 receptor antagonists (Yang, 2015).

### 3.5 Aconiti Radix

Aconiti Radix is the dry root of *Aconitum carmichaelii* Debx, pertaining to Ranunculaceae, which is acrid, bitter and hot in nature (State Pharmacopoeia Commission, 2020; Zhang and Ye, 2020). Aconiti Radix mainly contains alkaloids such as aconitine, hypaconitine, and mesaconitine. Yu et al. (2020a) found that hypaconitine could induce TRPV4-mediated calcium influx, causing an increase in cell temperature, and speculated that hypaconitine may have the ability to activate TRPV4 channels. They suggested that the “hot/cold” properties of TCM can be verified from the perspectives of cellular and molecular biology, providing a method by which to study the theory of TCM properties.

## 4 Studies on regulation of transient receptor potential channels by warm traditional Chinese medicines and their components

### 4.1 Asari Radix et rhizoma

Asari Radix et Rhizoma is the dry root and rhizoma of *Asarum heterotropoldes* Fr.Schmidt var. *mandshuricum (Maxim.) Kitag*, *Asarum sieboldii* Miq. var. *seoulense* Nakai or *Asarum sieboldii* Miq, pertaining to Aristolochiaceae, which is acrid and warm in nature (State Pharmacopoeia Commission, 2020; Zhang and Ye, 2020). Yu detected the main ingredients in the water extract of Asarum (WA), such as higenamine and caulesnarinside, *via* the UHPLC-Q-TOF/MS method, and in a capsaicin-induced pain experiment, observed that WA inhibited the number of foot lifts and the time of foot licking in mice. This suggests that WA may inhibit pain induced by acute pain experiments using TRPV1 (Yu, 2019). The volatile oil is the main active ingredient of Asari Radix et Rhizoma, and is largely retained by the ethanol extract of TCM. Using an electrophysiological whole-cell patch-clamp technique and mouse hot and cold plate experiments, Liu et al. found that the transmembrane currents induced by ethanol extract of Asari Radix et Rhizoma were highly similar to those induced by TRPV1 agonist Caps in terms of current density and current-voltage relationships. They also found that ethanol extract of Asari Radix et Rhizoma can prolong the behavioral latency of cold and hot pain in mice, depending on dose. These results suggest that the volatile oil contained in the ethanol extract of Asari Radix et Rhizoma may activate TRPV1 channels (Liu et al., 2021).

### 4.2 Zingiberis Rhizoma Recens

Zingiberis Rhizoma Recens is the fresh rhizoma of *Zingiber officinale* Rosc, pertaining to Zingiberaceae, which is acrid and slightly warm in nature (State Pharmacopoeia Commission, 2020; Zhang and Ye, 2020). Yin showed that the three pungent compounds from Zingiberis Rhizoma Recens were mTRPV1 agonists, with an order of activation intensity of 6-shogaol > 6-gingerol > zingerone, while the molecular structure of 6-shogaol was most similar to that of capsaicin (Yin et al., 2019b). Yue et al. conducted experiments using the rat spine and showed that allyl isothiocyanate, an agonist of TRPA1, inhibited the action of zingerone, but not capsaicin. Zingerone, like TRPA1 agonist, can reduce the amplitude of the excitatory postsynaptic current induced by a single synapse. This indicates that the effect of zingerone enhances glutamatergic spontaneous excitatory transmission in rat substantia gelatinosa neurons is achieved by activating the TRPA1 channels, but not the TRPV1 channels (Yue et al., 2013).

### 4.3 Angelicae Dahuricae Radix

Angelicae Dahuricae Radix is the dry root of *Angelica dahurica* (Fisch.ex Hoffm.) Benth.et Hook.f. or *Angelica dahurica* (Fisch. ex Hoffm.) Benth.et Hook.f.var. formosana (Boiss.) State Pharmacopoeia Commission, (2020), Zhang and Ye, (2020), pertaining to Umbelliferae, which is acrid and warm in nature. Furanocoumarin imperatorin is one of the active ingredients of Angelicae Dahuricae Radix extracts. Chen et al. (2014) found that imperatorin could inhibit formalin and capsaicin-induced nociceptive responses, and confirmed that imperatorin could act as an agonist of TRPV1. This indicates that furanocoumarin imperatorin represents a novel class of TRPV1 regulators.

### 4.4 Caryophylli Flos

Caryophylli Flos is the dried flower bud of *Eugenia caryophllata* Thunb, pertaining to Myrtaceae, which is acrid and warm in nature (State Pharmacopoeia Commission, 2020). Inoue et al. used the blind whole-cell patch-clamp technique to demonstrate that eugenol activates TRPA1 channels in the nerve terminals, resulting in an increased spontaneous release of L-glutamate to substantia gelatinosa (SG) neurons. Eugenol therefore has the pharmacological effects of sedation and reduced threshold for convulsions (Inoue et al., 2012).

### 4.5 ChuanXiong rhizoma

ChuanXiong Rhizoma is the dried rhizoma of *Ligusticum chuanxiong* Hort, pertaining to Umbelliferae, which is acrid and warm in nature (State Pharmacopoeia Commission, 2020; Zhang and Ye, 2020). Tetramethylpyrazine is an alkaloid monomer extracted from ChuanXiong Rhizoma. In the study of the mechanism of the treatment of traumatic brain injury, Yu et al. (2020b) found that tetramethylpyrazine may regulate TRPM4 channels, aquaporin 4, and mitogen-activated protein kinase p38 by regulating the expression level of brain sulfonylurea receptor 1, with therapeutic effect.

### 4.6 Genkwa Flos

Genkwa Flos is the dried flower bud of *Daphne genkwa* Sieb. et Zucc, pertaining to Thymelaeaceae, which is bitter, acrid, warm, and toxic in nature (State Pharmacopoeia Commission, 2020; Zhang and Ye, 2020). Yin et al. (2019c) used 75% ethanol extract from Genkwa Flos to conduct animal behavioral experiments and transmembrane current detection in male C57BL/6 mice and HEK293 cells that expressed human TRPV1. The results showed that the latency of responses to cold or hot pain was prolonged in mice treated with Genkwa Flos, and hTRPV11/HEK293 cells could be activated by 10 g L^−1^ ethanol extract of Genkwa Flos to induce significant transmembrane current (*p* < 0.01). These findings demonstrated that the warm, anti-inflammatory and analgesic effects of Genkwa Flos may reflect a series of effects induced by the activation of TRPV1.

### 4.7 Artemisiae Argyi Folium

Artemisiae Argyi Folium is the leaf of *Artemisia argyi* Lévl.et Vant, pertaining to Compositae, which is acrid, bitter, warm, and slightly toxic in nature (State Pharmacopoeia Commission, 2020; Zhang and Ye, 2020). Guo et al. (2019) proposed that the ethanol extract of Artemisiae Argyi Folium contains ingredients that can activate TRPV1 channels. Using a whole-cell patch-clamp experiment, they showed that 10 mg/ml ethanol extract of Artemisiae Argyi Folium could activate TRPV1 to exceed activation generated by 10 μM capsaicin. It is suggested that the high capacity of Artemisiae Argyi Folium to activate TRPV1 channels, warm the meridian and dissipate cold to relieve pain effects of Artemisiae Argyi Folium may be related to the activation of TRPV1 channels.

### 4.8 Notopterygii Rhizoma et radix

Notopterygii Rhizoma et Radix is the dry rhizome and root of *Notopterygium incisum* Ting ex H.T.Chang or *Notopterygium franchetii* H. de Boiss, pertaining to Apiaceae, which is acrid, bitter, and warm in nature (State Pharmacopoeia Commission, 2020; Zhang and Ye, 2020). Liu et al. used the whole-cell voltage-clamp technique to record the effect of water extract of Notopterygii Rhizoma et Radix on transmembrane current. They found that the maximum transmembrane current produced by the water extract was at a concentration of 100 mg/ml, and that the current was completely abolished by capsaicin, a specific TRPV1 antagonist. Thus, they hypothesized that Notopterygii Rhizoma et Radix may underlie effects of pain relief, releasing exterior, and dissipating cold after activating TRPV1 channels (Liu et al., 2017a). Wang et al. studied the effect of water extract of Notopterygii Rhizoma et Radix on TRPV1 channels through a neuropathic pain model induced by acute pain and chronic constriction injury. They found that the extract significantly inhibited the mRNA and protein expression of TRPV1, thereby alleviating neuropathic pain (Wang et al., 2021).

### 4.9 Cnidii Fructus

Cnidii Fructus is the mature dry fruit of *Cnidium monieri* (L.) Cuss, pertaining to Umbelliferae, which is acrid, bitter, warm, and slightly toxic in nature (State Pharmacopoeia Commission, 2020; Zhang and Ye, 2020). Osthole is the bioactive ingredient of Cnidii Fructus. Neuberger et al. found that two types of osthole binding sites in the transmembrane region of TRPV3 were consistent with the binding sites of agonist 2-APB, indicating that osthole may be used to treat a variety of skin-related diseases (Neuberger et al., 2021b). Sun et al. found that osthole could significantly reduce scratching behavior induced by acetone-ether-water or histamine in mice, whereas mice with TRPV3 gene deletion showed no apparent response. This suggests that osthole can inhibit the function of TRPV3 and provide a therapeutic option for itch-related diseases (Sun et al., 2018). It has also been reported that limonene, an ingredient in the volatile oil of Cnidii Fructus, can activate the TRPA1 channels and exert an analgesic effect (Kaimoto et al., 2016). An et al. (2018) used the water and ethanol extract of Cnidii Fructus to detect the transmembrane currents of hTRPV1/HEK293 cells. The results showed that both the water and ethanol extracts of Cnidii Fructus could produce transmembrane currents in such cells, which suggests that it may exert its acrid and warm natures by activating the TRPV1 channels.

### 4.10 Allii Sativi Bulbus

Allii Sativi Bulbus is the bulb of *Allium sativum* L, pertaining to Liliaceae, which is acrid and warm in nature (State Pharmacopoeia Commission, 2020). Allicin from Allii Sativi Bulbus is an antioxidant. Tsuchiya et al. studied the regulatory effect of allicin on electrogenic ion transport in rat intestine *via* the transmural potential difference, and found that AP-18, an inhibitor of TRPA1 channels, significantly reduced the transmural potential difference induced by allicin. Allicin leads to electrogenic chloride and bicarbonate secretion *via* TRPA1. In contrast, in the absence of chloride and bicarbonate, the transmural potential difference in the colon is reduced. This may be related to the contractile function of the intestine (Tsuchiya and Kawamata, 2019).

### 4.11 Cinnamomi Ramulus

Cinnamomi Ramulus is the tender twig of *Cinnamomum cassia* Presl, pertaining to Lauraceae, which is acrid, sweet, and warm in nature (State Pharmacopoeia Commission, 2020; Zhang and Ye, 2020). Cinnamomi Ramulus contains cinnamaldehyde, which can desensitize both TRPV1 channels and cross-desensitize TRPA1 channels. Hu et al. (2014) indicated that the use of Cinnamomi Ramulus can block overactive bladder syndrome caused by abnormal activation of TRPV1 channels.

### 4.12 Murrayae Folium et cacumen

Murrayae Folium et Cacumen is the leaf and tender twig with leaves of *Murraya exotica* L. or *Murraya paniculate* (L.) Jack, pertaining to Rutaceae, which is acrid, slightly bitter, warm, and slightly toxic in nature (State Pharmacopoeia Commission, 2020). Two new natural coumarin enantiomers, namely B304-1 and B304-2, were extracted and isolated from the root of Murrayae Folium et Cacumen by Zhou, who verified that TRPV2 channels were highly expressed in brown adipocytes. Zhou et al. (2020) performed whole-cell patch-clamp experiments, the results of which showed that both B304-1 and B304-2 could inhibit endogenous TRPV2 currents in differentiated brown adipocytes, with potential for the treatment of energy imbalance or metabolic diseases.

## 5 Studies on regulation of transient receptor potential channels by cold traditional Chinese medicines and their components

### 5.1 Rhei Radix et rhizoma

Rhei Radix et Rhizoma is the root and rhizoma of *Rheum palmatum* L, *Rheum tanguticum* Maxin.ex Balf. or *Rheum officinale* Baill, pertaining to Polygonaceae, which is bitter and cold in nature (State Pharmacopoeia Commission, 2020; Zhang and Ye, 2020). Wan et al. found that Rhei Radix et Rhizoma significantly decreased the expression levels of TRPV1 and significantly increased those of TRPM8 in the hypothalamus and dorsal root ganglia using immunohistochemistry and Western blot methods. As a result, the yeast-induced body temperature rise in rats was significantly reduced (Wan et al., 2014).

### 5.2 Coptidis Rhizoma

Coptidis Rhizoma is the rhizoma of *Coptis chinensis* Franch, *C. deltoidea C. Y. Cheng et* Hsiao or *C. teeta* Wall, pertaining to Ranunculaceae, which is bitter and cold in nature (State Pharmacopoeia Commission, 2020; Zhang and Ye, 2020). Wan et al. found that after administration of Coptidis Rhizoma in rats, body temperature reduced, the expression level of TRPV1 mRNA decreased and that of TRPM8 mRNA in the hypothalamus and dorsal root ganglia was effectively increased. These results show that Coptidis Rhizoma can effectively improve the fever symptoms in an animal model of heat syndrome (Wan et al., 2014).

### 5.3 Macleayae cordatae herba

Macleayae cordatae Herba is the root and whole plant of *Macleaya cordata* (Willd.) R. Br. or *M. cordata* (Maxim.) Fedde, pertaining to Papaveraceae, which is bitter and cold in nature (Xu et al., 2018). The main chemical ingredients of Macleayae cordatae Herba are alkaloids. Sanguinarine is a benzylisoquinoline alkaloid extracted from Macleayae cordatae Herba, which has a wide range of pharmacological activities, such as anti-microbial, anti-tumor, anti-platelet, and anti-hypertensive (Li et al., 2021; Xu et al., 2021). Chi et al. used electrophysiological methods to demonstrate potent activation of TRPA1 channels by sanguinarine with EC_50_0.09 (0.04–0.13) μM, providing evidence that sanguinarine is an agonist of TRPA1 channels (Chi et al., 2021). This may lay the foundation for development of targeted drugs that modulate TRPA1 channel.

### 5.4 Paeoniae Radix Alba

Paeoniae Radix Alba is the dried root of *Paeonia lactiflora* pall, pertaining to Ranunculaceae, which is bitter, sour and slightly cold in nature (State Pharmacopoeia Commission, 2020; Zhang and Ye, 2020). Using Western blot, Zhang showed the effect of Paeoniae Radix Alba on TRP in DRG neurons and found that Paeoniae Radix Alba could restore the expression levels of TRPV1 and TRPM8 to normal levels, thus relieving chronic constriction injury of the sciatic nerve (Zhang, 2020).

### 5.5 Kansui Radix

Kansui Radix is the root tuber of *Euphorbia kansui* T.N.Liou ex T.P.Wang, pertaining to Euphorbiaceae, which is bitter, cold and poisonous in nature (State Pharmacopoeia Commission, 2020; Zhang and Ye, 2020). Han et al. found that ethanol extract of Kansui Radix could activate hTRPV1/HEK293 cells and induce clear transmembrane currents using an electrophysiological whole-cell patch-clamp technique. This indicates that Kansui Radix contains at least one clear TRPV1 agonist, and its anti-inflammatory and analgesic effects may be related to the activation of TRPV1 ion channels (Han et al., 2020).

### 5.6 Polygoni Cuspidati Rhizoma et radix

Polygoni Cuspidati Rhizoma et Radix is the root and rhizoma of *Polygonum cuspidatum* Sieb. et Zucc, pertaining to Polygonaceae, which is slightly bitter and slightly cold in nature (State Pharmacopoeia Commission, 2020; Zhang and Ye, 2020). Resveratrol (RESV) is abundant in Polygoni Cuspidati Rhizoma et Radix, and is a potent antioxidant with protective effects. Çiğ et al. found that TRPM2 channels play an important role in RESV protection of cells from bisphenol A (BisPH-A)-induced oxidative damage. RESV can also effectively reduce the cytotoxicity induced by BisPH-A, such as intracellular Ca^2+^ overload, reactive oxygen species generation, and caspase activations (Çiğ and Yildizhan, 2020; Dai et al., 2022).

## 6 Studies on regulation of transient receptor potential channels by cool traditional Chinese medicine and their components

### 6.1 Menthae Haplocalycis Herba

Menthae Haplocalycis Herba is the dry aboveground part of *Mentha haplocalyx* Briq, pertaining to Labiatae, which is acrid and cool in nature (State Pharmacopoeia Commission, 2020; Zhang and Ye, 2020). The monoterpenes menthol is an agonist or antagonist of TRPV1, TRPV3, TRPM8, and TRPA1 channels. Nguyen et al. found that the conserved arginine and glycine residues in the S4-S5 linker of rTRPV1 and mTRPV3 are required for menthol-evoked activation, which may provide a new avenue of research on the pain-relieving properties of menthol (Nguyen et al., 2021). Wang et al. found that carvacrol, an ingredient of peppermint oil, stimulates cell proliferation through TRPV3-mediated calcium influx. In addition, inhibition of TRPV3 expression can eliminate the promotion of carvacrol on the release of TGFα, as well as increasing the phosphorylation levels of EGFR, PI3K, and NF-κB. They also found that inhibition of TRPV3 channels can abolish carvacrol-induced skin diseases such as epidermal hyperplasia, providing a potential form of treatment for such diseases (Wang et al., 2021). Using patch clamp experiments, Niu et al. found that carvacrol can bind to and activate TRPV3 *via* the S2-S3 linker, providing evidence of chemical stimulation of the TRPV3 channel *via* this linker (Niu et al., 2022). Nazıroğlu found that carvacrol is a blocker of TRPM2 and TRPV4, and its blocking effect can be achieved by regulating oxidative stress and apoptosis in SH-SY5Y neuronal cells (Nazıroğlu, 2022).

## 7 Studies on regulation of transient receptor potential channels by neutral traditional Chinese medicine and their components

### 7.1 Sinomenii Caulis

Sinomenii Caulis is the dried ratan of *Sinomenium acutum* (Thunb.) Rehd. et Wils. or *Sinomenium acutum* (Thunb.) Rehd. et Wils. var. *cinereum* Rehd. et Wils, pertaining to Menispermaceae, which is bitter, acrid, and neutral in nature (State Pharmacopoeia Commission, 2020). Ma et al. found that sinomenine, a constituent of Sinomenii Caulis, significantly reduced capsaicin-induced cough in guinea pigs by reducing the calcium expression of SOX5 and TRPV1 and reducing the secretion of substance P (SP) and neurokinin A (Ma et al., 2021).

### 7.2 Rhodiolae Crenulatae Radix et rhizoma

Rhodiolae Crenulatae Radix et Rhizoma is the dried root and rhizoma of *Rhodiola crenulate* (Hook. f. et Thoms.) H. Ohba, pertaining to Crassulaceae, which is bitter, sweet, and neutral in nature (State Pharmacopoeia Commission, 2020). Salidroside is an effective ingredient of Rhodiolae Crenulatae Radix et Rhizoma. Li et al. found that salidroside was able to inhibit the expression level of TRPM8 by reducing the activity of cAMP response element-binding protein, thereby affecting the production of cold-induced mucin and protecting individuals from cold attacks (Li et al., 2013).

### 7.3 Folium Steviae

Folium Steviae is the leaf of *Stevia rebaudiana* (Bertoni) Hemsl, pertaining to Compositae, which is sweet and neutral in nature (Li, 2017). Steviol glycosides are natural, zero-calorie, high-intensity sweeteners derived from Folium Steviae. Philippaert et al. (2017) found that these glycosides potentiate the activity of TRPM5 channels and enhance glucose-induced insulin secretion, thus indicating that they can prevent and treat type 2 diabetes by regulating TRPM5 channels.

### 7.4 Scorpio

Scorpio is the dry bulk of *Buthus martensii* Karsch, pertaining to Buthidae, which is acrid, neutral and toxic in nature (State Pharmacopoeia Commission, 2020; Zhang and Ye, 2020). Hakim et al. isolated a peptide (BmP01) which can induce pain in mice by activating TRPV1 channels, from the venoms of Scorpio in TRPV1 gene knockout mice. The discovery of BmP01 provides possibilities for the treatment of pain caused by scorpion envenomation (Hakim et al., 2015; Jiang, 2015).

### 7.5 Cannabis Fructus

Cannabis Fructus is the ripe fruit of *Cannabis sativa* L, pertaining to Moraceae, which is sweet and neutral in nature (State Pharmacopoeia Commission, 2020; Zhang and Ye, 2020). Cannabidiol (CBD) is the active ingredient isolated from Cannabis sativa. It has been reported that CBD is associated with TRPV1, TRPV2, and TRPA1 channels (Iannotti et al., 2014). Kowalski et al. found that the excitatory effect of CBD on vagal afferent neurons may be related to TRPA1 (Kowalski et al., 2020). Pumroy et al. found that CBD interacts with TRPV2 channels through a hydrophobic pocket that is located between S5-S6 helices of adjacent subunits, providing a reference for the study of treatment options for glioblastoma multiforme (Pumroy et al., 2019). Inspired by the fact that TRPV2 and TRPV4 have very similar gene sequences, Huang et al. found that CBD interacts with residues on the S6 helix of TRPV4, thus suggesting that TRPV4 plays an important role in CBD inhibition of glioma (Huang et al., 2021). Maggi et al. confirmed using cell growth and other assays that CBD can inhibit the proliferation and cycle of chronic myeloid leukemia cells through the TRPV2 channel (Maggi et al., 2022).

## 8 Conclusion and perspectives

**FIGURE 2:**
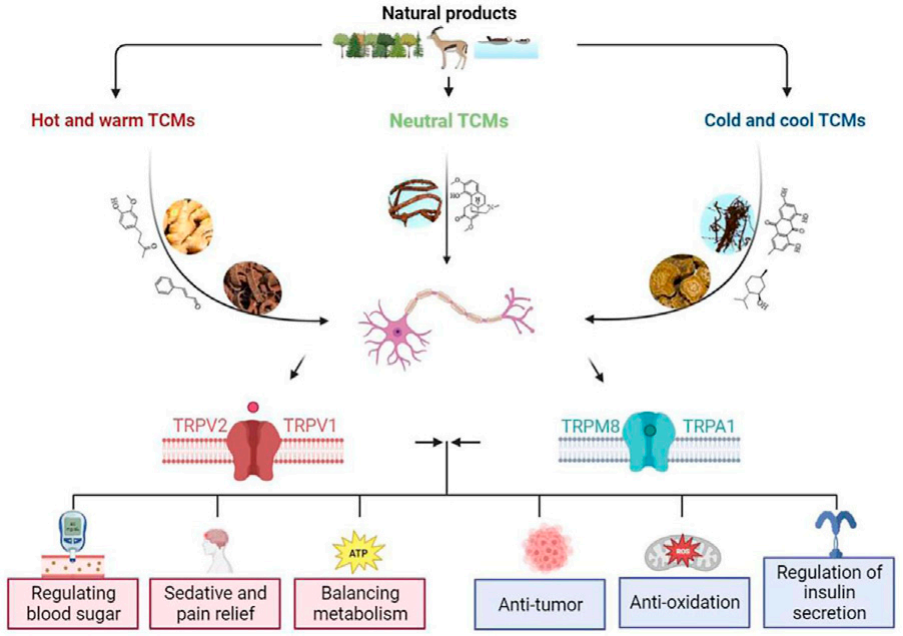
TCM modulates TRP channels to produce different pharmacological effects.

In this review, the effects of TCMs and their active ingredients on TRP ion channels are discussed including anti-inflammatory, analgesic, antihypertensive, hypoglycemic, and other pharmacological effects. Despite continued research progress on the protective effects of these TCMs by regulating TRP ion channels, the main active ingredients and mechanisms of their efficacy by activating or inhibiting TRP ion channels remain to be further studied. The information related to these TCMs and their components acting on different TRP channels are comprehensively summarized in Figure 2 and Tables 2, 3.

**TABLE 2:** List of Chinese herbal medicines that act on TRP channels

Name of the medicine	Medicinal properties	Effect	Clinical application	Channels of action	References
Capsici Fructus	Hot	Warm the center and dissipate cold, promote appetite, and digestion	Cold stagnation and abdominal pain, vomiting, diarrhea, pernio	TRPV1	(Caterina et al., 1997; State Pharmacopoeia Commission, 2020)
Cinnamomi Cortex	Hot	Supplement fire and assist Yang, dissipate cold and relieve pain, warm and unblock the channels, return fire to its source	Yang deficiency syndrome, pain syndromes due to congealing cold, cold congealing, and blood stasis	TRPV1, TRPM8, TRPA1	(Bhave et al., 2002; Ranasinghe et al., 2013; Hynkova et al., 2016; Li et al., 2016; State Pharmacopoeia Commission, 2020; Zhang and Ye, 2020)
Evodiae Fructus	Hot	Dissipate cold and relieve pain, direct counterflow downward and arrest vomiting, assist Yang and arrest diarrhea	All pains due to cold congealing in the liver meridian, vomiting and acid regurgitation, diarrhea due to deficiency-cold	TRPV1, TRPA1	(Iwaoka et al., 2016; State Pharmacopoeia Commission, 2020; Zhang and Ye, 2020; Li et al., 2016)
Zingiberis Rhizoma	Hot	Warm the center and dissipate cold, restore Yang to unblock the vessels, warm the lung, and dissolve rheum	Chills and pain of stomach cavity and abdomen, vomiting and diarrhea, Yang collapse syndrome, cough and panting due to cold fluid-retention	TRPV1, TRPM8	(Li et al., 2018; State Pharmacopoeia Commission, 2020; Zhang and Ye, 2020)
Aconiti Radix	Hot	Dispel wind and eliminate dampness, warm the meridians and relieve pain	Bì syndrome, cold pain in the heart and abdomen, and pain due to cold hernia	TRPV4	(Yu et al., 2020a; State Pharmacopoeia Commission, 2020; Zhang and Ye, 2020)
Asari Radix et Rhizoma	Warm	Release exterior and dissipate cold, expel wind and relieve pain, unblock the orifices, warm the lung and dissolve fluid retention	Wind-cold exterior syndrome, headache, toothache, wind-dampness impediment pain, allergic, rhinitis, sinusitis, blocked nose with discharge	TRPV1	(Yu, 2019; State Pharmacopoeia Commission, 2020; Zhang and Ye, 2020)
Zingiberis Rhizoma Recens	Slightly warm	Release exterior and dissipate cold, warm the middle and arrest vomiting, dissolve phlegm and relieve cough, remove toxic of fish and crab	Wind-cold exterior syndrome, vomiting due to stomach cold, cold-phlegm cough, intoxication by eating fish or crab	TRPV1, TRPA1	(Yue et al., 2013; Yin et al., 2019b; State Pharmacopoeia Commission, 2020; Zhang and Ye, 2020)
Angelicae Dahuricae Radix	Warm	Release exterior and dissipate cold, dispel wind and relieve pain, diffuse and unblock the nasal orifices, dry dampness and arrest vaginal discharge, resolve swelling and expel pus	Wind-cold exterior syndrome, headache, pain in supra-orbital bone, toothache, and wind-damp impediment pain, allergic rhinitis, sinusitis, blocked nose with discharge, leukorrhea, sores, and ulcers with swelling and pain	TRPV1	(Chen et al., 2014; State Pharmacopoeia Commission, 2020)
Caryophylli Flos	Warm	Warm the center and direct counterflow downward, nourishing kidney and assist Yang	Deficient cold of spleen and stomach, hiccup vomiting, poor appetite, vomiting and diarrhea, cold pain in the heart and abdomen, impotence due to kidney deficiency	TRPA1	(Inoue et al., 2012; State Pharmacopoeia Commission, 2020; Zhang and Ye, 2020)
ChuanXiong Rhizoma	Warm	Invigorate blood and move qi, dispel wind and alleviate pain	Pains due to qi stagnation and blood stasis, headache, Bì syndrome	TRPM4	(Yu et al., 2020b; State Pharmacopoeia Commission, 2020; Zhang and Ye, 2020; Zhao et al., 2021)
Genkwa Flos	Warm	Expel fluid retention by drastic purgation, kill worms and cure sores (for external use)	Edema, accumulated water in the chest and abdomen, and accumulation of phlegm, scabies, tinea, favus, swollen carbuncle, and pernio	TRPV1	(Yin et al., 2019c; State Pharmacopoeia Commission, 2020; Zhang and Ye, 2020
Artemisiae Argyi Folium	Warm	Warm channels and staunch bleeding, dissipate cold to relieve pain, dispel dampness and relieve itching for external use	Bleeding due to deficiency-cold, irregular menstruation, painful menstruation and threatened miscarriage, itchy skin	TRPV1	(Guo et al., 2019; State Pharmacopoeia Commission, 2020; Zhang and Ye, 2020)
Notopterygii Rhizoma et Radix	Warm	Release exterior and dissipate cold, dispel wind and dampness, relieve pain	External-contraction of wind-cold complicated by dampness, wind-cold-damp impediment, and pain in shoulders and back	TRPV1	(Liu et al., 2017a; State Pharmacopoeia Commission, 2020; Zhang and Ye, 2020)
Cnidii Fructus	Warm	Kill worms and relieve itching, dry dampness and dispel wind, warm the kidney and strengthen Yang	Pudendum itching, eczema and pruritus, as well as acariasis, impotence due to kidney deficiency and sterility due to uterus-cold, leukorrhea due to cold dampness, and lumbago caused by the damp-bì syndrome	TRPV1, TRPV3, TRPA1	(Kaimoto et al., 2016; An et al., 2018; State Pharmacopoeia Commission, 2020; Neuberger et al., 2021b)
Allii Sativi Bulbus	Warm	Resolve toxins and relieve edema, kill worms, arrest dysentery	Carbuncle, swelling, sore and toxic, scabies, phthisis, paroxysmal cough, diarrhea, dysentery	TRPA1	(Tsuchiya and Kawamata, 2019; State Pharmacopoeia Commission, 2020; Zhang and Ye, 2020)
Cinnamomi Ramulus	Warm	Induce sweating to release muscles, warm and unblock meridians, reinforce Yang and promote qi transformation, lower downflow of reversed qi	Wind-cold exterior syndrome, pains induced by wind-cold-damp arthralgia, palpitation phlegm-fluid retention, edema, up-rushing of qi (running piglet qi)	TRPV1, TRPA1	(Hu et al., 2014; State Pharmacopoeia Commission, 2020; Zhang and Ye, 2020)
Murrayae Folium et Cacumen	Warm	Activate qi and relieve pain, invigorate blood and disperse stasis	Stomach-ache, pains of rheumatism and arthralgia, toothache, swelling and pain from falls, insect or snake bite	TRPV2	(State Pharmacopoeia Commission, 2020; Zhou et al., 2020)
Rhei Radix et Rhizoma	Cold	Attack accumulation by purgation, clear heat and drain fire, cool blood and resolve toxic, expel stasis and promote menstruation flow, drain dampness and relieve jaundice	Constipation due to stagnation of excess heat, red eyes and swollen throat, gum pain, hematemesis due to blood heat, pyocutaneous diseases, intestinal abscess with abdominal pain, burn and scald, blood stasis, jaundice, and stranguria	TRPV1, TRPM8	(Wan et al., 2014; SState Pharmacopoeia Commission, 2020; Zhang and Ye, 2020)
Coptidis Rhizoma	Cold	Clear heat and dry damp, reduce fire and remove toxic	Stuffiness and fullness due to damp-heat, vomiting, diarrhea, and dysentery, the exuberance of heart-fire syndrome, exuberance of stomach fire syndrome, carbuncle, abscess, furuncle, sores, swollen and painful eyes, eczema, purulent discharge of an auditory canal	TRPV1, TRPM8	(Wan et al., 2014; State Pharmacopoeia Commission, 2020; Zhang and Ye, 2020)
Macleayae cordatae Herba	Cold	Disperse stasis and relieve edema, dispel wind and resolve toxins, kill worms and relieve itching	Thyroid neoplasm, cutaneous tumor, carbuncle, swelling, furuncle, injuries from falls, pains of rheumatism and arthralgia, neurodermatitis, poisonous insect bite	TRPA1	(Xu et al., 2018; Chi et al., 2021)
Paeoniae Radix Alba	Slightly cold	Nourish blood and regulate menstruation, astring yin and arrest sweating, soften the liver and relieve pain, calm and subdue liver Yang	Blood deficiency, a syndrome caused by hyperactivity of liver Yang, rib-side and abdominal pain, spastic pain in four limbs, night sweating, and spontaneous sweating	TRPV1, TRPM8	(State Pharmacopoeia Commission, 2020; Zhang, 2020; Zhang and Ye, 2020)
Kansui Radix	Cold	Expel fluid retention by drastic purgation, relieve swelling and dissipate masses	Edema, accumulated water in the chest and abdomen, and accumulation of phlegm rheum, epilepsy with wind-phlegm, sores and carbuncles with swelling and toxic	TRPV1	(Han et al., 2020; State Pharmacopoeia Commission, 2020; Zhang and Ye, 2020)
Polygoni Cuspidati Rhizoma et Radix	Slightly cold	Remove dampness and jaundice, clear heat and remove toxic, remove blood stasis and relieve pain, remove phlegm and stop cough	Blood stasis syndrome, dampness-heat jaundice, turbid stranguria, leukorrhagia, carbuncle, swelling, sore, scald and snake bite, cough due to lung heat	TRPM2	(State Pharmacopoeia Commission, 2020; ÇÇiğ and Yildizhan, 2020; Zhang and Ye, 2020)
Menthae Haplocalycis Herba	Cool	Scatter and dissipate wind-heat, clear head and eyes, soothe throat, promote eruption, soothe liver and move qi	Wind-heat exterior syndrome and warm diseases at the early stage, wind-heat headache and red-eye with profuse tears, sort throat and pain, measles failing to erupt, itching rubella, syndrome of liver depression, and qi stagnation	TRPV1, TRPV3, TRPV4, TRPM2, TRPM8, TRPA1	(State Pharmacopoeia Commission, 2020; Zhang and Ye, 2020; Nguyen et al., 2021; Nazıroğlu, 2022; Niu et al., 2022)
Sinomenii Caulis	Neutral	Dispel wind and dampness, unblock the meridians and collaterals, promote diuresis	Pains of rheumatism and arthralgia, joint swelling, palsy itching	TRPV1	(State Pharmacopoeia Commission, 2020; Ma et al., 2021)
Rhodiolae Crenulatae Radix et Rhizoma	Neutral	Supplement qi and invigorate blood circulation, unblock the collaterals and relieve panting	Qi deficiency and blood stasis, chest pain, wind-stroke to hemiplegia, lassitude and asthma	TRPM8	(Li et al., 2013; State Pharmacopoeia Commission, 2020)
Folium Steviae	Neutral	Promoting fluid and relieving thirst, promote diuresis for antihypertensive effect	Diabetes, hypertension	TRPM5	(Li, 2017a; Philippaert et al., 2017)
Scorpio	Neutral	Extinguish wind and suppress convulsion, unblock the collaterals and relieve pain, reduce toxicity and dissipate masses	Spasm and convulsion, wind-damp obstinate impediment, hemilateral and overall headache, sores and pyogenic infections, scrofula and phlegm node	TRPV1	(Hakim et al., 2015; State Pharmacopoeia Commission, 2020; Zhang and Ye, 2020)
Cannabis Fructus	Neutral	Moisten the intestines to promote defecation	Constipation due to dryness of intestine	TRPV1, TRPV2, TRPV4, TRPA1	(Iannotti et al., 2014; State Pharmacopoeia Commission, 2020; Zhang and Ye, 2020; Huang et al., 2021a)

**TABLE 3:** List of compounds from Chinese herbal medicine that can affect TRP channels

Compounds	Structural formula	Origins	References
Capsaicin (CAP)	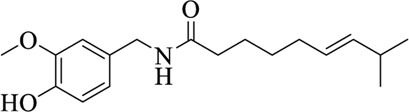	Capsici Fructus	Caterina et al. (1997)
Cinnamaldehyde (CA)	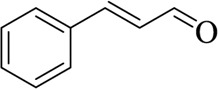	Cinnamomi Cortex	Li et al. (2016)
Evodiamine	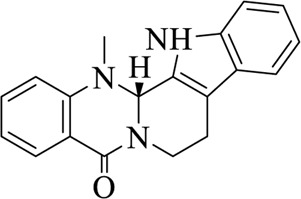	Evodiae Fructus	Iwaoka et al. (2016)
Rutaecarpine (Rut)	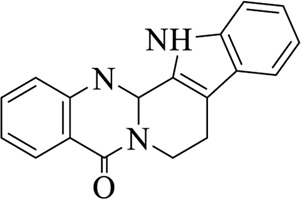	Evodiae Fructus	Wang et al. (2016)
6-gingerol	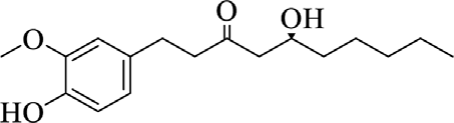	Zingiberis Rhizoma, Zingiberis Rhizoma Recens	Yang, (2015)
10-gingerol	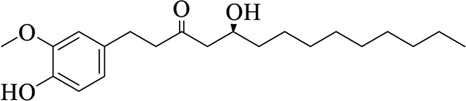	Zingiberis Rhizoma, Zingiberis Rhizoma Recens	Yang, (2015)
Hypaconitine	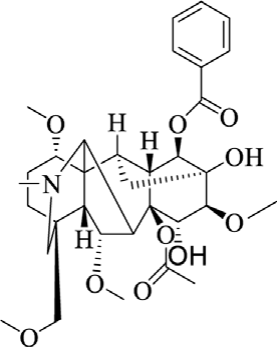	Aconiti Radix	Yu et al. (2020a)
Higenamine	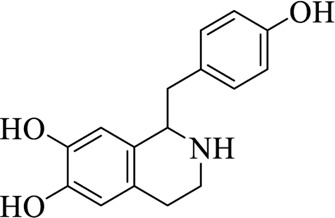	Asari Radix et Rhizoma	Yu, (2019)
6-shogaol	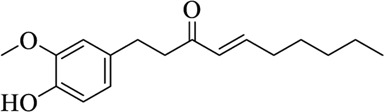	Zingiberis Rhizoma, Zingiberis Rhizoma Recens	Yin et al. (2019b)
Zingerone	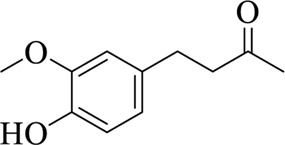	Zingiberis Rhizoma, Zingiberis Rhizoma Recens	Yin et al. (2019b)
Imperatorin	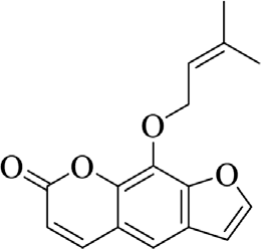	Angelicae Dahuricae Radix	Inoue et al. (2012)
Eugenol	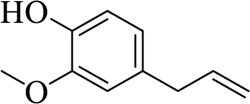	Caryophylli Flos	Inoue et al. (2012)
Tetramethylpyrazine	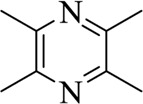	ChuanXiong Rhizoma	Yu et al. 2020b
Osthole	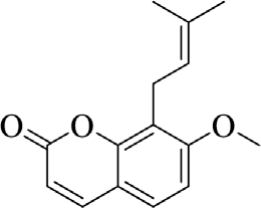	Cnidii Fructus	Neuberger et al. 2021b
Limonene	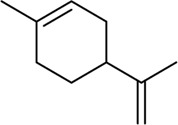	Cnidii Fructus	Kaimoto et al. (2016)
Allicin	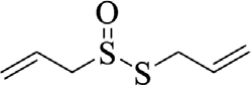	Allii Sativi Bulbus	Tsuchiya and Kawamata. (2019)
B304-1	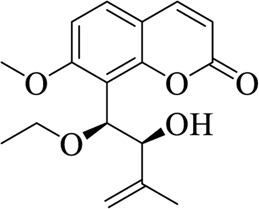	Murrayae Folium et Cacumen	Zhou et al. (2020)
B304-2	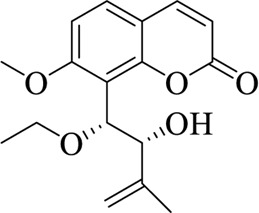	Murrayae Folium et Cacumen	Zhou et al. (2020)
Sanguinarine	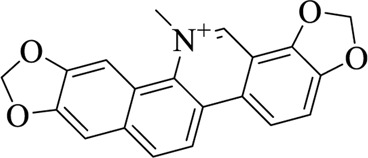	Macleayae cordatae Herba	Xu et al. (2021)
Resveratrol (RESV)	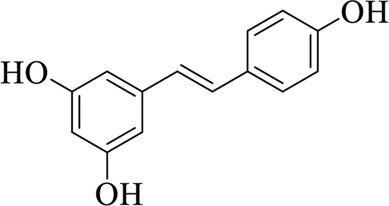	Polygoni Cuspidati Rhizoma et Radix	Dai et al. (2022)
Menthol	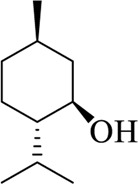	Menthae Haplocalycis Herba	Nguyen et al. (2021)
Carvacrol	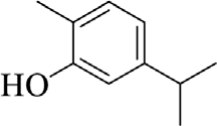	Menthae Haplocalycis Herba	Wang et al. (2021b)
Sinomenine	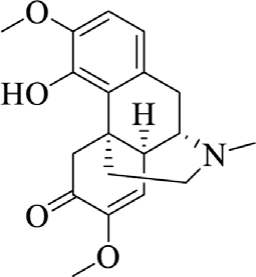	Sinomenii Caulis	Ma et al. (2021)
Salidroside	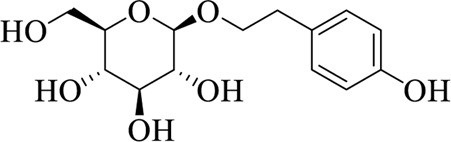	Rhodiolae Crenulatae Radix et Rhizoma	Li et al. (2013)
Stevioside	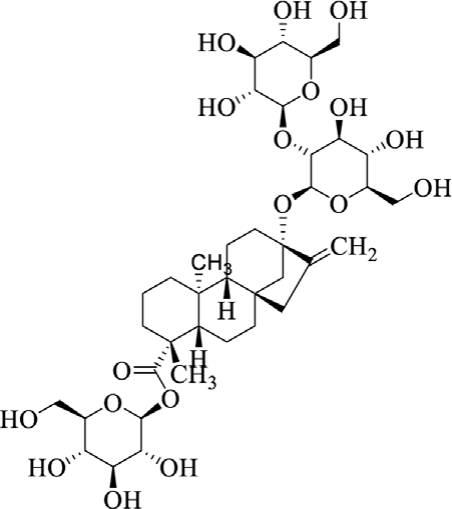	Folium Steviae	Philippaert et al. (2017)
Cannabidiol (CBD)	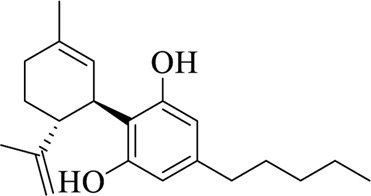	Cannabis Fructus	Iannotti et al. (2014)

For example, some of the TCMs discussed in this review have been investigated using only water or ethanol extracts, and further studies on major active ingredients will be performed in the future. In addition, some TCMs only show effects on TRP ion channels, while the molecular mechanism of regulation needs to be further explored.

In conclusion, we hope that this review will provide a reference for further research on the development and utilization of TRP ion channels in TCM, and provide new research ideas for the study of cold, hot, warm, cool, and neutral natures of TCM.

## Abbreviations

BisPH-A, Bisphenol A; CA, Cinnamaldehyde; CAP, Capsaicin; CBD, Cannabidiol; CCI, Chronic constriction injury; PKA, Protein kinase; RESV, Resveratrol; SG, Substantia gelatinosa; SGs, Steviol glycosides; TCM, Traditional Chinese medicine; TM, Transmembrane spanning domains; TRIM4, Tripartite motif-containing 4; TRP, Thermal transient receptor potential; TRPA1, Transient receptor potential ankyrin 1; TRPM2, Transient receptor potential melastatin 2; TRPM4, Transient receptor potential melastatin 4; TRPM5, Transient receptor potential melastatin 5; TRPM8, Transient receptor potential melastatin 8; TRPV1, Transient receptor potential vanilloid 1; TRPV2, Transient receptor potential vanilloid 2; TRPV3, Transient receptor potential vanilloid 3; TRPV4, Transient receptor potential vanilloid 4; WA, Water extract of Asarum; WN, Water extract of Notopterygii Rhizoma et Radix.
