# Supplementary material for: Regulation of transient receptor potential channels by traditional Chinese medicines and their active ingredients
Source: Front Pharmacol. 2022 Oct 13;13:1039412. doi: 10.3389/fphar.2022.1039412 (PMC9606675; doi:10.3389/fphar.2022.1039412)
Supplement: Supplementary file 1 [file Table1.DOCX]

Supplementary Material

# Supplementary Figures


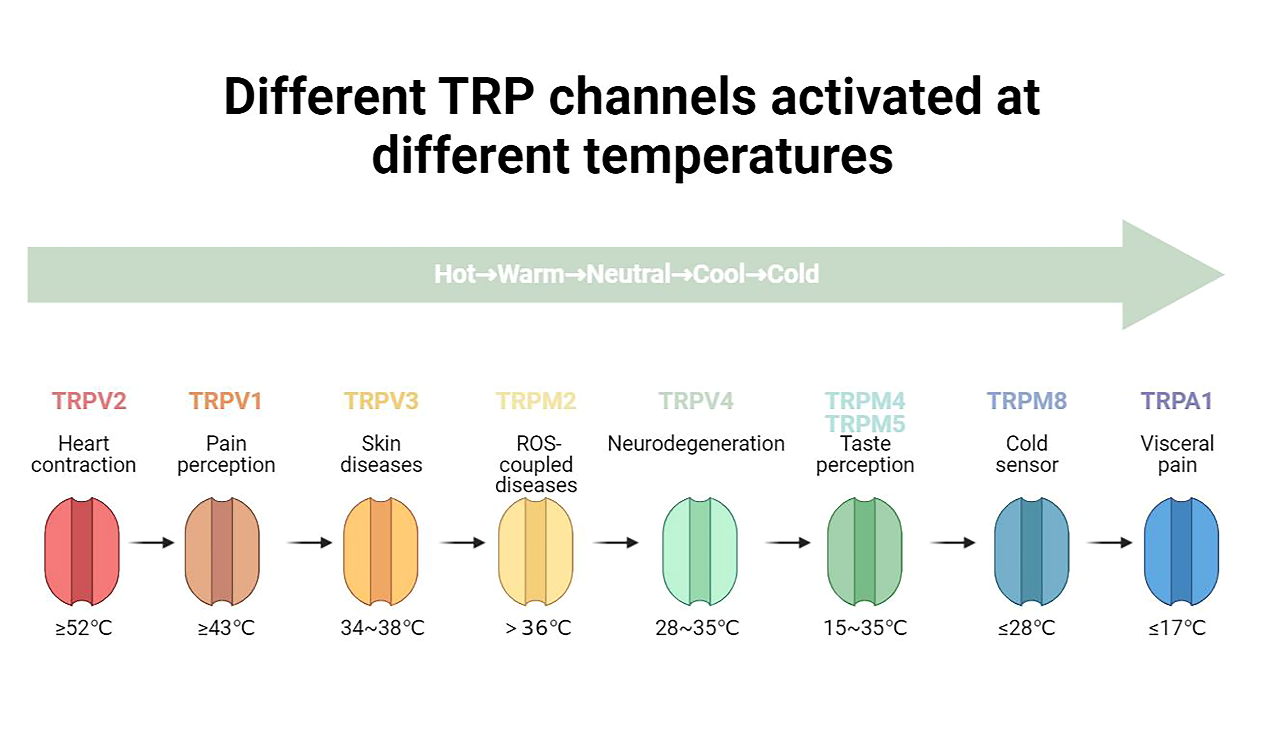
 **Supplementary Figure 1.** **Different TRP channels activated at different temperatures**

**
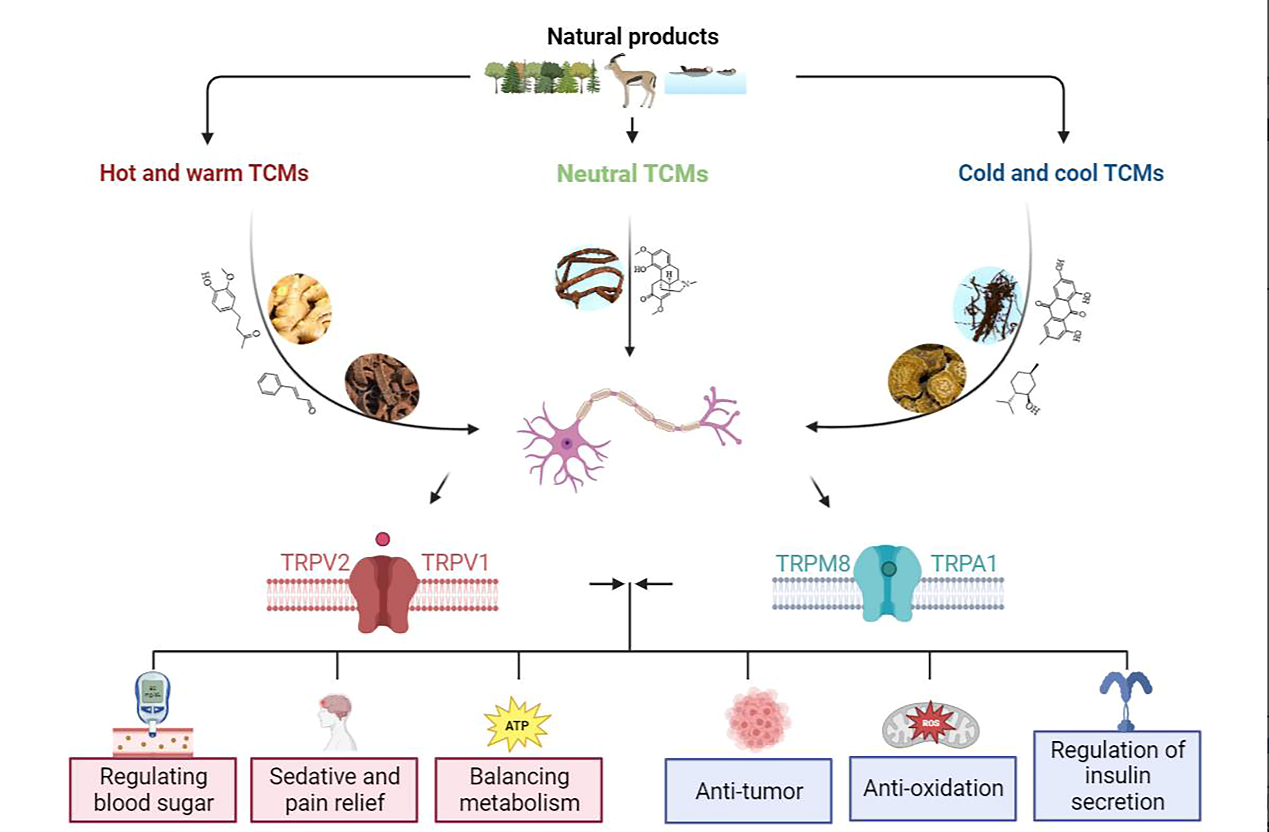
**

**Supplementary Figure 2.** **TCM modulates TRP channels to produce different pharmacological effects**

# Supplementary Tables

**Table 1. Introduction to the TRP channel**

| Channel | Temperature sensitivity | Main tissue distribution | Functions | References |
| --- | --- | --- | --- | --- |
| TRPV1 | ≥43℃ | Sensory neurons, brain, skin. | Noxious thermoreceptors; also involved in inflammatory pain, thermal nociceptive hypersensitivity, hippocampal chronic depression, obesity, diabetes, bladder function, hypertension, hypothermia, gastroenteritis, renal excretory function | (Vay et al., 2012; Uchida et al., 2017; Wu, 2019) |
| TRPV2 | ≥52℃ | Sensory neurons, brain, spinal cord, lung, liver, spleen, colon, heart, immunocyte. | Extreme temperature sensor; innate immune system | (Kochukov et al., 2006; Vay et al., 2012; Naticchioni et al., 2015; Uchida et al., 2017) |
| TRPV3 | 34℃-38℃ | Sensory neurons, skin, brain, spinal cord, stomach, colon. | Warmth receptors; may be associated with detection of harmful heat levels | (Kochukov et al., 2006; Vay et al., 2012; Uchida et al., 2017; Neuberger et al., 2021a) |
| TRPV4 | 28℃-35℃ | Sensory neurons, skin, brain, kidneys, lungs, inner ear, bladder. | Thermoreceptors; may be associated with noxious mechanical pain and thermal nociceptive sensitization | (Vay et al., 2012; Uchida et al., 2017; Huang et al., 2019) |
| TRPM2 | >36℃ | Brain, immunocyte, pancreas. | Thermoreceptors; associated with pain due to inflammation, diabetes, tumors, cardiovascular system | (Kahya et al., 2017; Uchida et al., 2017) |
| TRPM4 | 15℃-35℃ | Heart, liver, immunocyte, pancreas. | Thermoreceptors; associated with inflammation-induced pain, tumors, cardiovascular system | (Talavera et al., 2005; Uchida et al., 2017; Dutta Banik et al., 2018) |
| TRPM5 | 15℃-35℃ | Taste cells, pancreas. | Warmth receptors; may be related to insulin secretion, obesity, diabetes | (Talavera et al., 2005; Uchida et al., 2017; Dutta Banik et al., 2018) |
| TRPM8 | ≤28℃ | Sensory neurons, bladder. | Nontoxic cold sensory receptors, behavioral thermoregulation, cold-mediated analgesia; cold injurious sensation in some neurons | (Vay et al., 2012; Uchida et al., 2017; Yin et al., 2018) |
| TRPA1 | ≤17℃ | Sensory neurons, heart, lungs, brain, pancreas, gastrointestinal tract, bladder. | Cold, mechanically- and chemically-induced injuries, cold nociceptive sensitization | (Vay et al., 2012; Uchida et al., 2017) |

**Table 2. List of Chinese herbal medicines that act on TRP channels**

| Name of the medicine | Medicinal properties | Effect | Clinical application | Channels of action | References |
| --- | --- | --- | --- | --- | --- |
| Capsici Fructus | Hot | Warm the center and dissipate cold, promote appetite, and digestion | Cold stagnation and abdominal pain, vomiting, diarrhea, pernio | TRPV1 | (Caterina et al., 1997; State Pharmacopoeia Commission, 2020) |
| Cinnamomi Cortex | Hot | Supplement fire and assist Yang, dissipate cold and relieve pain, warm and unblock the channels, return fire to its source | Yang deficiency syndrome, pain syndromes due to congealing cold, cold congealing, and blood stasis | TRPV1, TRPM8, TRPA1 | (Bhave et al., 2002; Ranasinghe et al., 2013; Hynkova et al., 2016; Li et al., 2016; State Pharmacopoeia Commission, 2020; Zhang and Ye, 2020) |
| Evodiae Fructus | Hot | Dissipate cold and relieve pain, direct counterflow downward and arrest vomiting, assist Yang and arrest diarrhea | All pains due to cold congealing in the liver meridian, vomiting and acid regurgitation, diarrhea due to deficiency-cold | TRPV1, TRPA1 | (Iwaoka et al., 2016; State Pharmacopoeia Commission, 2020; Zhang and Ye, 2020; Li et al.) |
| Zingiberis Rhizoma | Hot | Warm the center and dissipate cold, restore Yang to unblock the vessels, warm the lung, and dissolve rheum | Chills and pain of stomach cavity and abdomen, vomiting and diarrhea, Yang collapse syndrome, cough and panting due to cold fluid-retention | TRPV1, TRPM8 | (Li et al., 2018; State Pharmacopoeia Commission, 2020; Zhang and Ye, 2020) |
| Aconiti Radix | Hot | Dispel wind and eliminate dampness, warm the meridians and relieve pain | *Bì* syndrome, cold pain in the heart and abdomen, and pain due to cold hernia | TRPV4 | (State Pharmacopoeia Commission, 2020; Yu et al., 2020a; Zhang and Ye, 2020) |
| Asari Radix et Rhizoma | Warm | Release exterior and dissipate cold, expel wind and relieve pain, unblock the orifices, warm the lung and dissolve fluid retention | Wind-cold exterior syndrome, headache, toothache, wind-dampness impediment pain, allergic, rhinitis, sinusitis, blocked nose with discharge | TRPV1 | (Yu, 2019; State Pharmacopoeia Commission, 2020; Zhang and Ye, 2020) |
| Zingiberis Rhizoma Recens | Slightly warm | Release exterior and dissipate cold, warm the middle and arrest vomiting, dissolve phlegm and relieve cough, remove toxic of fish and crab | Wind-cold exterior syndrome, vomiting due to stomach cold, cold-phlegm cough, intoxication by eating fish or crab | TRPV1, TRPA1 | (Yue et al., 2013; Yin et al., 2019b; State Pharmacopoeia Commission, 2020; Zhang and Ye, 2020) |
| Angelicae Dahuricae Radix | Warm | Release exterior and dissipate cold, dispel wind and relieve pain, diffuse and unblock the nasal orifices, dry dampness and arrest vaginal discharge, resolve swelling and expel pus | Wind-cold exterior syndrome, headache, pain in supra-orbital bone, toothache, and wind-damp impediment pain, allergic rhinitis, sinusitis, blocked nose with discharge, leukorrhea, sores, and ulcers with swelling and pain | TRPV1 | (Chen et al., 2014; State Pharmacopoeia Commission, 2020; Zhang and Ye, 2020) |
| Caryophylli Flos | Warm | Warm the center and direct counterflow downward, nourishing kidney and assist Yang | Deficient cold of spleen and stomach, hiccup vomiting, poor appetite, vomiting and diarrhea, cold pain in the heart and abdomen, impotence due to kidney deficiency | TRPA1 | (Inoue et al., 2012; State Pharmacopoeia Commission, 2020) |
| ChuanXiong Rhizoma | Warm | Invigorate blood and move qi, dispel wind and alleviate pain | Pains due to qi stagnation and blood stasis, headache, *Bì* syndrome | TRPM4 | (State Pharmacopoeia Commission, 2020; Yu et al., 2020b; Zhang and Ye, 2020; Zhao et al., 2021) |
| Genkwa Flos | Warm | Expel fluid retention by drastic purgation, kill worms and cure sores (for external use) | Edema, accumulated water in the chest and abdomen, and accumulation of phlegm, scabies, tinea, favus, swollen carbuncle, and pernio | TRPV1 | (Yin et al., 2019c; State Pharmacopoeia Commission, 2020; Zhang and Ye, 2020) |
| Artemisiae Argyi Folium | Warm | Warm channels and staunch bleeding, dissipate cold to relieve pain, dispel dampness and relieve itching for external use | Bleeding due to deficiency-cold, irregular menstruation, painful menstruation and threatened miscarriage, itchy skin | TRPV1 | (Guo et al., 2019; State Pharmacopoeia Commission, 2020; Zhang and Ye, 2020) |
| Notopterygii Rhizoma et Radix | Warm | Release exterior and dissipate cold, dispel wind and dampness, relieve pain | External-contraction of wind-cold complicated by dampness, wind-cold-damp impediment, and pain in shoulders and back | TRPV1 | (Liu et al., 2017a; State Pharmacopoeia Commission, 2020; Zhang and Ye, 2020) |
| Cnidii Fructus | Warm | Kill worms and relieve itching, dry dampness and dispel wind, warm the kidney and strengthen Yang | Pudendum itching, eczema and pruritus, as well as acariasis, impotence due to kidney deficiency and sterility due to uterus-cold, leukorrhea due to cold dampness, and lumbago caused by the damp-*bì* syndrome | TRPV1, TRPV3, TRPA1 | (Kaimoto et al., 2016; An et al., 2018; State Pharmacopoeia Commission, 2020; Zhang and Ye, 2020; Neuberger et al., 2021b) |
| Allii Sativi Bulbus | Warm | Resolve toxins and relieve edema, kill worms, arrest dysentery | Carbuncle, swelling, sore and toxic, scabies, phthisis, paroxysmal cough, diarrhea, dysentery | TRPA1 | (Tsuchiya and Kawamata, 2019; State Pharmacopoeia Commission, 2020) |
| Cinnamomi Ramulus | Warm | Induce sweating to release muscles, warm and unblock meridians, reinforce Yang and promote qi transformation, lower downflow of reversed qi | Wind-cold exterior syndrome, pains induced by wind-cold-damp arthralgia, palpitation phlegm-fluid retention, edema, up-rushing of qi (running piglet qi) | TRPV1, TRPA1 | (Hu et al., 2014; State Pharmacopoeia Commission, 2020; Zhang and Ye, 2020) |
| Murrayae Folium et Cacumen | Warm | Activate qi and relieve pain, invigorate blood and disperse stasis | Stomach-ache, pains of rheumatism and arthralgia, toothache, swelling and pain from falls, insect or snake bite | TRPV2 | (State Pharmacopoeia Commission, 2020; Zhou et al., 2020) |
| Rhei Radix et Rhizoma | Cold | Attack accumulation by purgation, clear heat and drain fire, cool blood and resolve toxic, expel stasis and promote menstruation flow, drain dampness and relieve jaundice | Constipation due to stagnation of excess heat, red eyes and swollen throat, gum pain, hematemesis due to blood heat, pyocutaneous diseases, intestinal abscess with abdominal pain, burn and scald, blood stasis, jaundice, and stranguria | TRPV1, TRPM8 | (Wan et al., 2014; State Pharmacopoeia Commission, 2020; Zhang and Ye, 2020) |
| Coptidis Rhizoma | Cold | Clear heat and dry damp, reduce fire and remove toxic | Stuffiness and fullness due to damp-heat, vomiting, diarrhea, and dysentery, the exuberance of heart-fire syndrome, exuberance of stomach fire syndrome, carbuncle, abscess, furuncle, sores, swollen and painful eyes, eczema, purulent discharge of an auditory canal | TRPV1, TRPM8 | (Wan et al., 2014; State Pharmacopoeia Commission, 2020; Zhang and Ye, 2020) |
| Macleayae cordatae Herba | Cold | Disperse stasis and relieve edema, dispel wind and resolve toxins, kill worms and relieve itching | Thyroid neoplasm, cutaneous tumor, carbuncle, swelling, furuncle, injuries from falls, pains of rheumatism and arthralgia, neurodermatitis, poisonous insect bite | TRPA1 | (Xu et al., 2018; Chi et al., 2021) |
| Paeoniae Radix Alba | Slightly cold | Nourish blood and regulate menstruation, astring yin and arrest sweating, soften the liver and relieve pain, calm and subdue liver Yang | Blood deficiency, a syndrome caused by hyperactivity of liver Yang, rib-side and abdominal pain, spastic pain in four limbs, night sweating, and spontaneous sweating | TRPV1, TRPM8 | (State Pharmacopoeia Commission, 2020; Zhang, 2020; Zhang and Ye, 2020) |
| Kansui Radix | Cold | Expel fluid retention by drastic purgation, relieve swelling and dissipate masses | Edema, accumulated water in the chest and abdomen, and accumulation of phlegm rheum, epilepsy with wind-phlegm, sores and carbuncles with swelling and toxic | TRPV1 | (Han et al., 2020; State Pharmacopoeia Commission, 2020; Zhang and Ye, 2020) |
| Polygoni Cuspidati Rhizoma et Radix | Slightly cold | Remove dampness and jaundice, clear heat and remove toxic, remove blood stasis and relieve pain, remove phlegm and stop cough | Blood stasis syndrome, dampness-heat jaundice, turbid stranguria, leukorrhagia, carbuncle, swelling, sore, scald and snake bite, cough due to lung heat | TRPM2 | (State Pharmacopoeia Commission, 2020; Çiğ and Yildizhan, 2020; Zhang and Ye, 2020) |
| Menthae Haplocalycis Herba | Cool | Scatter and dissipate wind-heat, clear head and eyes, soothe throat, promote eruption, soothe liver and move qi | Wind-heat exterior syndrome and warm diseases at the early stage, wind-heat headache and red-eye with profuse tears, sort throat and pain, measles failing to erupt, itching rubella, syndrome of liver depression, and qi stagnation | TRPV1, TRPV3, TRPV4, TRPM2, TRPM8, TRPA1 | (State Pharmacopoeia Commission, 2020; Zhang and Ye, 2020; Nguyen et al., 2021; Nazıroğlu, 2022; Niu et al., 2022) |
| Sinomenii Caulis | Neutral | Dispel wind and dampness, unblock the meridians and collaterals, promote diuresis | Pains of rheumatism and arthralgia, joint swelling, palsy itching | TRPV1 | (State Pharmacopoeia Commission, 2020; Ma et al., 2021) |
| Rhodiolae Crenulatae Radix et Rhizoma | Neutral | Supplement qi and invigorate blood circulation, unblock the collaterals and relieve panting | Qi deficiency and blood stasis, chest pain, wind-stroke to hemiplegia, lassitude and asthma | TRPM8 | (Li et al., 2013; State Pharmacopoeia Commission, 2020) |
| Folium Steviae | Neutral | Promoting fluid and relieving thirst, promote diuresis for antihypertensive effect | Diabetes, hypertension | TRPM5 | (Li, 2017a; Philippaert et al., 2017) |
| Scorpio | Neutral | Extinguish wind and suppress convulsion, unblock the collaterals and relieve pain, reduce toxicity and dissipate masses | Spasm and convulsion, wind-damp obstinate impediment, hemilateral and overall headache, sores and pyogenic infections, scrofula and phlegm node | TRPV1 | (Hakim et al., 2015; State Pharmacopoeia Commission, 2020; Zhang and Ye, 2020) |
| Cannabis Fructus | Neutral | Moisten the intestines to promote defecation | Constipation due to dryness of intestine | TRPV1, TRPV2, TRPV4, TRPA1 | (Iannotti et al., 2014; State Pharmacopoeia Commission, 2020; Zhang and Ye, 2020; Huang et al., 2021a) |

**Table 3 List of compounds from Chinese herbal medicine that can affect TRP channels**

| Compounds | Structural formula | Origins | References |
| --- | --- | --- | --- |
| Capsaicin (CAP) |  | Capsici Fructus | (Caterina et al., 1997) |
| Cinnamaldehyde (CA) |  | Cinnamomi Cortex | (Li et al., 2016) |
| Evodiamine |  | Evodiae Fructus | (Iwaoka et al., 2016) |
| Rutaecarpine (Rut) |  | Evodiae Fructus | (Wang et al., 2016) |
| 6-gingerol |  | Zingiberis Rhizoma, Zingiberis Rhizoma Recens | (Yang, 2015) |
| 10-gingerol |  | Zingiberis Rhizoma, Zingiberis Rhizoma Recens | (Yang, 2015) |
| Hypaconitine |  | Aconiti Radix | (Yu et al., 2020a) |
| Higenamine |  | Asari Radix et Rhizoma | (Yu, 2019) |
| 6-shogaol |  | Zingiberis Rhizoma, Zingiberis Rhizoma Recens | (Yin et al., 2019b) |
| Zingerone |  | Zingiberis Rhizoma, Zingiberis Rhizoma Recens | (Yin et al., 2019b) |
| Imperatorin |  | Angelicae Dahuricae Radix | (Chen et al., 2014) |
| Eugenol |  | Caryophylli Flos | (Inoue et al., 2012) |
| Tetramethylpyrazine |  | ChuanXiong Rhizoma | (Yu et al., 2020b) |
| Osthole |  | Cnidii Fructus | (Neuberger et al., 2021b) |
| Limonene |  | Cnidii Fructus | (Kaimoto et al., 2016) |
| Allicin |  | Allii Sativi Bulbus | (Tsuchiya and Kawamata, 2019) |
| B304-1 |  | Murrayae Folium et Cacumen | (Zhou et al., 2020) |
| B304-2 |  | Murrayae Folium et Cacumen | (Zhou et al., 2020) |
| Sanguinarine |  | Macleayae cordatae Herba | (Xu et al., 2021) |
| Resveratrol (RESV) |  | Polygoni Cuspidati Rhizoma et Radix | (Dai et al., 2022) |
| Menthol |  | Menthae Haplocalycis Herba | (Nguyen et al., 2021) |
| Carvacrol |  | Menthae Haplocalycis Herba | (Wang et al., 2021b) |
| Sinomenine |  | Sinomenii Caulis | (Ma et al., 2021) |
| Salidroside |  | Rhodiolae Crenulatae Radix et Rhizoma | (Li et al., 2013) |
| Stevioside |  | Folium Steviae | (Philippaert et al., 2017) |
| Cannabidiol (CBD) |  | Cannabis Fructus | (Iannotti et al., 2014) |
